# Uterus Didelphys: Report of a Puerperal Torsion and a Review of the Literature

**DOI:** 10.1155/2012/190167

**Published:** 2012-08-02

**Authors:** Lucio M. A. Cipullo, Slobodan Milosavljevic, Elisabeth D. van Oudgaarden

**Affiliations:** Department of Obstetrics and Gynaecology, Slotervaart Medisch Centrum, Louwesweg 6, 1066 EC Amsterdam, The Netherlands

## Abstract

A 29-year-old Para 2 was admitted to the emergency department with increasing lower abdominal pain. The patient had undergone an uncomplicated elective repeat caesarean section 7 days before being admitted to the emergency department. An emergency laparotomy revealed a uterus didelphys with a torsion of one of the uteri.

## 1. Introduction

Uterine torsion is the twist of the uterus between the cervix and uterine body. A minor degree of rotation of the pregnant uterus is fairly common during the third trimester of pregnancy but is deemed rather negligible. In contrast, an axial rotation of more than 45 degrees is quite unusual and its occurrence is defined as uterine torsion. The unusual occurrence of this latter condition during the puerperal period is often associated with predisposing factors altering the shape or position of the uterus or adnexa.

A severe torsion of the uterus can cause irreversible ischemic injury or serious thrombotic accidents. Hence, it is crucial to diagnose this condition quickly.

In this paper we describe a case of a uterine torsion in a woman carrying a Müllerian anomaly (*uterus didelphys*). The torsion involved one of the uteri and occurred on the seventh day after an uncomplicated caesarean section for which it was necessary to perform a surgical reintervention. 

## 2. Case Presentation

A 29-year-old woman Para 2 was admitted to the emergency department with increasing lower abdominal pain. The patient had no history of systemic disease or chronic abdominal tenderness. Her past obstetric history revealed a caesarean section performed at 38 weeks because of a breech presentation three years earlier. 

The patient had undergone an uncomplicated elective repeat caesarean section 7 days before being admitted to the emergency department. Indication for CS was breech presentation at term. The operation was without complications except some heavy bleeding occurred after the incision of the lower uterine segment; the bleeding was controlled by means of haemostatic stitches. The postoperative course was uneventful and the patient was discharged in good clinical conditions four days after CS.

Three days after CS she reported a significant decrease of postpartum discharge (*lochia*) as well as a sudden complete stop of vaginal bleeding and discharge on day 4 after SC. 

On admission, patient was alert, conscious, and well-oriented. She complained of severe low abdominal pain. Blood pressure was 140/80 mm Hg, HR 105 b/min, and temp. 37.4. The pain started the day before the admission as an abdominal discomfort with a sudden increase during the following day. At the time of her admission the patient described a constant low abdominal/pelvic pain with colicky exacerbations. Examination at this stage found no vaginal bleeding. Palpation revealed a painful swelling on the right side just above the pelvis. There was no sign of peritoneal irritation or abdominal distension. The wound seemed to be healing well. Lab tests at admission: Hb 12,8 g/dL, Hct 35, PLT 280, and WBC 10,5 × 10^9^/L.

Routine urine examination was normal. Ultrasound investigation showed a strongly involuted uterus (LD 7.5 cm, TD 4.2 cm, APD 3.7 cm) which was localized centrally in the pelvis and also an oval-shaped mass on the right side of the lower abdomen (25 cm × 8 cm), showing thickened walls with complex internal echo patterns apparently suggesting a organized haematoma. In the pouch of Douglas, there were no signs of active bleeding or clots. Despite of the administration of meperidine hydrochloride 100 mg IM, a reassessment after two hours showed increased symptoms with additional signs of peritoneal irritation and restlessness whereupon an explorative laparotomy was decided. 

Access was gained through the previous Pfannenstiel incision. At the opening of the abdomen a large amount of coagulated, dark red blood was evident amongst the intestinal loops. A further exploration of the pelvis showed an involuted uterus without signs of recent hysterotomy. On the right side, at the level of right iliac fossa, there was an oval, reddish, soft structure with a maximum diameter 25 cm. Mobilisation and further inspection revealed an enlarged uterus situated on the right side in respect to the small one (*uterus didelphys*). This second uterus showed a torsion of 90° on its axis and had a congested appearance. 

**Figure 1:**
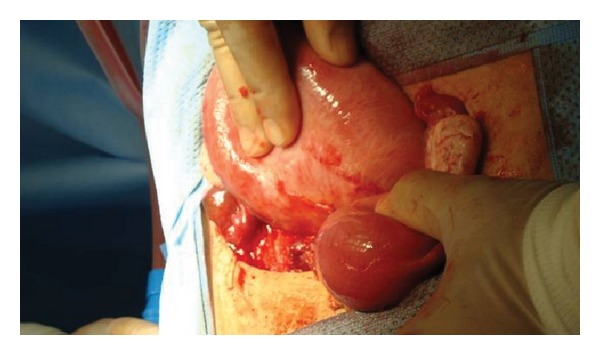
Uterus before detorsion.

**Figure 2:**
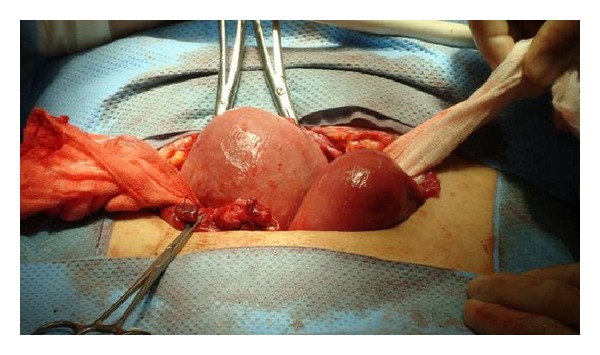
Uterus after detorsion.

The uterine cavity was filled with blood (*lochia*) coming out the tubae by squeezing of the uterus. There were no signs of uterine weakening. During the inspection before detorsion it was noted a transverse hysterotomy suture on the right side of the uterus which was not bleeding. The other smaller uterus appeared firmly fixed to the anterior pelvic fascia and the lower segment of the left anterolateral wall of the other uterus (Figure 1). Both adnexa were normal at inspection. All adhesions needed to be removed before attempting a complete emptying and detorsion of the organ (Figure 2). Postoperative recovery was uneventful and the patient could be discharged 5 days after the operation in good clinical condition. She got prescribed enoxaparin 20 mg per day for 6 weeks to prevent thromboembolism.

## 3. Discussion

Uterine torsion is the rotation of the uterus at the level of the lower uterine segment of more than 45 degrees along its axis. Dextrorotation occurs in two-thirds of the cases and laevorotation is found in the other one-third [1]. 

Labbe published the first case of uterine torsion in 1876 [2]. There have been very few cases since this first publication, all nearly exclusively regarding torsion of the uterus occurring during pregnancy. The torsion of the uterus during the puerperal period is even rarer with only three cases reported in literature to date.

The risk factors for uterine torsion during the puerperium include fixation of the uterus by adhesions, ovarian tumor, uterine myomas, large neoplasms, and uterine Müllerian anomalies. According to some authors intrinsic intrapelvic pathology is responsible for 66% of the cases during pregnancy [3]. A study conducted with MRI evaluation of patients following low transverse caesarean section suggested that, occasionally, poor healing of the hysterotomic scar may result in suboptimal restoration of normal cervical length and strength predisposing the uterus to torsion in those cases [4]. In the case presented here, the association of the two factors that were described determined the torsion: uterus didelphys together with iatrogenic adhesion between one of the uteri and the pelvic wall. The clinical presentation of puerperal uterine torsion is nonspecific and may differ from the symptoms of torsion in pregnancy. Symptoms at presentation could suggest an adnexal torsion or other colicky abdominal pain. The most common symptom is abdominal pain varying from mild abdominal tenderness through to symptoms of an acute abdomen making diagnosis difficult especially in the absence of MRI imaging. The sudden cessation of the physiological vaginal discharge associated with abdominal pain in puerperal period is strongly suggestive of uterine torsion. In about 11 percent of cases torsion is asymptomatic [5]. In case of torsion, a prompt diagnosis is crucial to start surgery as soon as possible to avoid the risk of ischemic and thromboembolic complications. Ultrasound is not specific for this kind of diagnosis. In some cases, if previous ultrasound scans revealed fibroids that have changed position, a torsion of a myomatous uterus may be suspected. Use of CT scan or MRI is a valid diagnostic tool when available in an emergency situation. MRI provides an accurate evaluation. The wall of the upper vagina changes from normal H configuration to an x-shaped configuration in uterine torsion [6, 7]. 

In the case discussed here, the differential diagnosis was made between an adnexal tumor, a partially organised bleeding or a retroperitoneal mass. Existence of a Müllerian anomaly was ignored. Only the direct examination revealed the presence of a double-uterus of large proportions twisted on its axis but also rotated behind the small uterus. Most likely, the first caesarean section had been carried out in the left uterus causing adhesions between the left side of the uterus and the anterior pelvic wall. Both the anomalous position of the uterus due to the firm adherences and the Müllerian anomaly increased the risk of torsion. During inspection of the rotated uterus, the hysterotomy commonly performed in a different area, appeared to be positioned rather abnormally on the right side of the organ. This important detail suggested that the uterus had undergone the torsion after caesarean section had been performed. The subsequent torsion determined both the abrupt cessation of the postpartum discharge of haematic lochia and over distension of the uterine body. Rotation of the uterus on its axis had partially involved the uterine vessels causing a general congestion of the organ. Accurate exploration of uterus excluded the presence of any irreversible ischemic damage, a condition which would require a hysterectomy. 

It is, however, very difficult to determine whether the ischemic injury affecting the uterus is reversible or not, because puerperal torsion is a rare pathological condition. 

Hysterectomy needs to be considered for women who have fulfilled reproductive wishes with uterine necrosis resulting from prolonged torsion [8]. Bilateral plication of the round ligaments can be done to prevent immediate postpartum recurrence of uterus torsion [9]. This may help to keep uterus in its natural position and reduce the effect of iatrogenic uterine adhesion. Bilateral plication of uterosacral ligaments is also described, which may provide resistance to torsion and prevent long-term recurrence of this condition [10].
